# Targeting Epigenetic Plasticity to Reduce Periodontitis-Related Inflammation in Diabetes: CBD, Metformin, and Other Natural Products as Potential Synergistic Candidates for Regulation? A Narrative Review

**DOI:** 10.3390/ijms26072853

**Published:** 2025-03-21

**Authors:** Amelia Tero-Vescan, Mark Slevin, Amalia Pușcaș, Dragoș Sita, Ruxandra Ștefănescu

**Affiliations:** 1 Department of Medical Chemistry and Biochemistry, Faculty of Medicine in English, George Emil Palade University of Medicine, Pharmacy, Science, and Technology of Târgu Mureș, 540139 Târgu Mureş, Romania; amelia.tero-vescan@umfst.ro; 2 Centre for Advanced Medical and Pharmaceutical Research, George Emil Palade University of Medicine, Pharmacy, Science, and Technology of Târgu Mureș, 540139 Târgu Mureş, Romania; 3 Department of Biochemistry and Chemistry of the Environmental Factors, Faculty of Pharmacy, George Emil Palade University of Medicine, Pharmacy, Science, and Technology of Târgu Mureș, 540139 Târgu Mureş, Romania; amalia.puscas@umfst.ro; 4 Department of Odontology and Oral Pathology, Faculty of Dental Medicine, George Emil Palade University of Medicine, Pharmacy, Science, and Technology of Târgu Mureș, 540139 Târgu Mureş, Romania; dragos.sita@umfst.ro; 5 Department of Pharmacognosy and Phytotherapy, Faculty of Pharmacy, George Emil Palade University of Medicine, Pharmacy, Science, and Technology of Târgu Mureș, 540139 Târgu Mureş, Romania; ruxandra.stefanescu@umfst.ro

**Keywords:** periodontitis, diabetes, epigenetics, cannabidiol

## Abstract

Periodontitis is unanimously accepted to be the sixth complication of diabetes mellitus (DM), while the inverse relationship of causality is still to be deciphered. Among the proposed mechanisms is gut dysbiosis, which is responsible for the systemic release of proinflammatory mediators. In this process, Gram-negative bacteria from the oral cavity enter the general circulation, leading to the emergence of bi-hormonal beta-pancreatic cells that lack the ability to secrete insulin. Additionally, epigenetic and adaptive mechanisms in affected cells may play a role in reducing inflammation. The release of reactive oxygen species, proinflammatory cytokines, and adipokines, such as interleukins, tumor necrosis factor alpha, leptin, prostaglandin E2, C-reactive protein, or matrix metalloproteinases, determine epigenetic changes, such as the methylation of DNA nucleotides or changes in the activity of histone acetylases/deacetylases. The management of periodontitis involves targeting inflammation, and its potential connection to epigenetic modulation observed in other chronic conditions may help to explain its role in preventing DM in affected patients. This review focuses on the key epigenetic changes in periodontitis that might contribute to DM development, and explores the mechanisms and novel multi-drug therapies that could help to prevent these effects.

## 1. Introduction

Both periodontitis and diabetes mellitus (DM) are widely spread chronic diseases among adults, with a strong, cause–effect interrelationship [1]. In 2021, the International Diabetes Federation reported that 10.5% of the adult population (aged between 20 and 79) have diabetes, with around half of these not aware of the condition. In addition, the upward trend in the incidence of type 2 diabetes cases indicates that, by 2045, the number of sufferers will be 783 million, a further increase of 43% [2]. Periodontitis affects 45% of young adults, with the incidence reaching 60% in those aged over 65, and is considered the sixth major consequence of DM [3,4]. Statistics show that patients with periodontitis have a 33% increased risk of hyperglycemia and a 1.24 times higher risk of developing DM in 5 years [5]. From a clinical perspective, managing DM patients with periodontitis is extremely challenging because both conditions are characterized by and share a chronic inflammatory and collagenolytic status, and, moreover, they potentiate each other. Current treatment strategies involve glycemic control and reducing the bacterial burden in periodontitis rather than reducing inflammation and resolving excessive collagenolysis [6].

Recently, a key role has been suggested for the epigenetic modulation of target genes predisposing to and informing disease progression in DM/periodontitis. Epigenetics is generally defined as a change in gene function induced by environmental factors, individual lifestyle, chemical and physical factors, or heritability, without producing changes in the sequence of nucleotides in DNA [7,8]. These changes involve DNA methyltransferases (DNMTs), the methylation of DNA nucleotides containing cytosine in a CpG sequence [9], or the post-translational acetylation, methylation, phosphorylation, citrullination, and ubiquitylation of histone [10,11].

A very recent article highlights the complex interactions between immune responses, metabolic alterations, oxidative stress, and epigenetic changes in orofacial pathology responsible for pain sensation signaling, and proposes redox homeostasis and epigenetic modulation of pain-related genes and nociceptive signaling. These mechanisms are particularly relevant in the context of diabetes and periodontitis, two interrelated conditions characterized by chronic inflammation, impaired immune function, and oxidative stress. Since the causative link between the two chronic pathologic conditions was already established, the question is whether metabolic reprogramming is possible, and which would be the potential candidates [12].

A novel potential regulator of epigenetic plasticity is cannabidiol (CBD), a dominant natural compound in the extract of *Cannabis sativa*, along with Δ9-tetrahydrocannabinol (THC) [13]. Unlike THC, CBD does not have a psychostimulatory effect and the tendency to abuse; therefore, it is used in neurological diseases and mental illnesses for its anticonvulsant, anxiolytic, and antipsychotic effects [14] in cardiovascular disease (CVD) [15], but also in conditions involving chronic inflammation and pain, infection, and cancer [16,17].

Whilst there are numerous studies that have evaluated the anti-inflammatory effect of CBD in periodontitis, the mechanisms by which it modulates epigenetic changes, considered at least partially responsible for the pathological degeneration in DM, to our knowledge, have not yet been elucidated.

## 2. Periodontitis as a Risk Factor for DM

### 2.1. Overview

Growing evidence suggests that chronic periodontitis is a significant risk factor for diabetes mellitus (DM), with a bidirectional relationship in which periodontitis can contribute to diabetes progression, while diabetic changes exacerbate periodontal inflammation. Although the exact mechanisms remain unclear, several pathways have been proposed.

Oral–gut dysbiosis, caused by changes in the oral microbiome, may alter intestinal immune responses, leading to systemic inflammation [18]. Epigenetic modifications in inflamed periodontal tissues may induce temporary cellular changes that contribute to long-term pathological conditions in DM [3,19]. Additionally, oral pathogens can enter systemic circulation through ulcerated periodontal membranes and directly affect pancreatic β-cells, thereby impairing insulin secretion [20].

### 2.2. Mechanistic Details

Histone acetylation is closely linked to gene expression regulation. Histone acetyltransferases (HATs) and histone deacetylases (HDACs) maintain histone acetylation homeostasis, playing a role in numerous pathological conditions [21].

Supporting this hypothesis, a study using ligature-induced periodontitis and Porphyromonas gingivalis gavage in C57BL/6 mice demonstrated increased DNA methyltransferase 3 beta (DNMT3b) expression in gut and maxilla tissue, as shown by the immunohistochemistry (IHC) [22]. Systemic DNMT3b overexpression (via miR-124-3p) led to excessive DNA methylation of the TRAF6 promoter, enhancing inflammation through TNF-α and IL-6 signaling, while also reducing cellular viability and increasing apoptosis [23]. Similarly, *P. gingivalis* exposure has been linked to β-cell apoptosis through caspase-3 and caspase-8 activation, along with reduced protection via Akt pathway inhibition [24].

Additionally, *P. gingivalis*-induced lipopolysaccharides (LPSs) have been shown to stimulate histone acetylation, upregulating inflammatory genes such as p300/CBP HAT and nuclear factor-kappa B (NF-κB) [25]. Concurrently, *P. gingivalis* reduces HDAC1 activity in human gingival epithelial and keratinocyte cell lines, further promoting acetylation-driven inflammation [26].

### 2.3. Further Evidence Linking DM to Modified Periodontal DNA Methylation and Vice Versa

Periodontitis is sometimes considered as the sixth complication/risk factor associated with DM; however, epigenetic modifications directly associated with the onset and progressive stages of diabetes can significantly impact upon the pathological development of periodontitis. An in vitro study performed on periodontal cells collected from patients with or without DM showed that a deficiency of the NAD+-dependent histone deacetylase, sirtuin-6 (SIRT6), led to the reduced resolution of inflammation, to the accumulation of apoptotic neutrophils, and to the modification of the efferocytosis process of macrophages by changing the levels of miR-216/217 and increasing the expression of CD36 and DEL-1, which could contribute to the progression or severity of periodontal disease in people with DM [27].

Li et al. (2021) showed that streptozotocin-induced DM in mini-pigs significantly changed the DNA methylation patterns of multiple genes in the periodontal tissue, resulting in changes in lipid transport, hormone secretion, upregulated inflammatory markers, and heightened immune response, angiogenesis, and metabolic status, all of which contribute to the development and aggravation of periodontitis [28]. In patients, hyperglycemia caused by DM was demonstrated to produce genetic changes in >1000 genes from gingival tissue. The study further identified a genetic polymorphism of RAGEG82S as a factor that exacerbates periodontitis in patients with DM [29,30].

Although limited, there is some confirmatory evidence of cell phenotypical change and protein modification associated with these neo-epigenetic profiles. For example, gut pathogens [released systemically] also induced epigenetic changes by decreasing another methyltransferase, DNMT-1, which induced the downregulation of the β-cell-specific transcription factors PDX1 and NK6 homeobox 1 (NKX6.1), and lead to the appearance of β-pancreatic bi-hormonal cells, which lost their ability to secrete insulin and other hormones, and which is associated with poorer glycemic control [31,32].

These studies strongly indicate a credible cyclical overlap between molecular switches and network pathways encompassing the two diseases, and, since projects such as ENCODE have recently fully mapped periodontal epigenetic changes, a complete list of mapped protein target alterations over time should allow for the characterization of the critical periodontal–DM axis cross-over over the coming years [7]. Currently known epigenetic modifications are presented in Table 1.

### 2.4. ROS as a Key Mechanistic Controller of Periodontitis-Associated DM

The presence of Gram-negative bacteria in the oral cavity leads to the release of endotoxins and LPSs, which, in turn, can generate ROS. In vitro studies show that the treatment of periodontal ligament fibroblasts (PDLF) with LPS exacerbates ROS production and promotes the binding of thioredoxin (TXNIP) and NOD-like receptor protein 3 (NLRP3) to form NLRP3 inflammasomes [36].

In periodontitis, neutrophils are considered the main source of ROS through the NADPH oxidase 2 (Nox2) pathway [38]. Numerous markers of oxidative stress are altered in patients with periodontitis. Specifically, the activity of superoxide dismutase (SOD) and catalase (CAT) is decreased, while local levels of parameters, such as the total antioxidant capacity (TAOC), malondialdehyde (MDA), glutathione peroxidase, nitric oxide, protein carbonyls, thiobarbituric acid-reacting substances, advanced oxidation protein products, lipid peroxidation products, total oxidative status, and 8-hydroxydeoxyguanosine, are increased [39,40].

ROS causes modifications in mitochondrial DNA and mitochondrial dysfunction, leading to excess ROS production [41]. ROS play an essential role in cell proliferation, migration, apoptosis, and wound healing. Thus, ROS production increases the expression of dynamin-related protein 1 (Drp1), responsible for mitochondrial fission, leading to mitochondrial dysfunction, decreased ATP levels, and apoptosis in human periodontal ligament stem cells (hPDLSCs) [42].

ROS and epigenetic change: ROS can directly influence gene transcription, and there is substantial evidence supporting their capacity to alter the epigenetic profile, wherein the source, amount, and length of time of ROS present in the cell can lead to different epigenetic outcomes. In diabetic conditions, the macrophage phenotype remains proinflammatory at M1 through epigenetic changes that occur during hematopoietic stem cell (HSC) differentiation in an ROS-dependent manner [43]. It has been shown that hematopoietic stem cells from high-fat-diet (HFD) or db/db mice exhibited significantly higher oxidative stress and a concomitant Nox2-dependent increase in DNA methyltransferase-1 (DNMT-1) expression. This led to the hypermethylation of Notch1, PU.1, and Klf4, which are crucial transcription factors blocking HSC differentiation into monocytes/macrophages. Decreased DNMT-1 in smokers with periodontitis, accompanied by the lower expression of Nox2/iNOS in tissue biopsies, indicated an ROS/oxidative stress-modulated epigenetic control over cell differentiation, phenotype, and function [44].

### 2.5. The Role of Proinflammatory Cytokines in Periodontitis as Epigenetic Modulators of DM

Neutrophils, in addition to their direct antimicrobial effects, determine the release of inflammatory mediators in response to danger signals caused by bacteria or tissue damage [45]. The LPSs released by the interaction of *P. gingivalis* with neutrophils in the host cell, in turn, trigger the release of proinflammatory cytokines, such as IL-1β and IL-6, or TNF-α and ROS, which aggravate the inflammatory response [46].

IL-1β primes/degranulates polymorphonuclear leukocytes, increases the synthesis of proinflammatory prostaglandins (PGs) and matrix metalloproteinases (MMPs), inhibits collagen synthesis, and activates T- and B-lymphocytes. IL-6 activates the production of inflammatory mediators, including PGE2, in gingival fibroblasts via inducing cyclooxygenase-2 in a tyrosine kinase pathway, while IL-6 stimulates the formation of osteoclasts responsible for bone resorption and facilitates the differentiation of T-cells [47].

IL-1β and PGE2 secreted in periodontitis complete this vicious circle, inducing epigenetic changes through modifying the expression of DNA methylating/demethylating enzymes and altering the patterns of DNA methylation. This could be a mechanism for the systemic dissemination of the local inflammatory condition [48].

Data from the literature indicate that patients with both DM and periodontitis, compared to those without DM, have significantly elevated levels of leptin and significantly reduced levels of adiponectin [49]. Adiponectin has an anti-inflammatory and insulin-sensitizing effect; it favors glucose uptake and the beta-oxidation of fatty acids for energy purposes by reducing NF-κB activation, by activating the AMPK and peroxisome proliferator-activated receptor alpha (PPAR-α) signaling pathways, while leptin has a proinflammatory effect by activating T-cells [50,51]. Pathogens such as *P. gingivalis*, *T. denticola*, and *Fusobacterium nucleatum* inhibit adiponectin and its receptor expression in periodontal cells [52]. Low levels of adiponectin cannot maintain inhibited NF-κB nuclear translocation, which increases insulin receptor substrate-1 (IRS-1) serine phosphorylation-producing insulin resistance [53].

Periodontitis independently changes the serum levels of leptin, adiponectin, and C-reactive protein (CRP) [54]. The mechanism by which leptin promotes periodontitis pathogenesis is still unknown; however, recent studies suggest that leptin induces proinflammatory M1 macrophage skewing and decreases M2 macrophage polarization. In vitro studies demonstrate that, at a molecular level, leptin activates the NOD-like receptor family pyrin domain-containing 3 (NLRP3) inflammasome [55]. A study conducted by Guo et al. shows that the effect of leptin is dose-dependent. At low doses, it increases the osteoprotegerin (OPG)/receptor activator of NF-κB ligand (RANKL) ratio in gingival fibroblasts, while high concentrations have the opposite effect. These mechanisms suggest that periodontitis produces the metabolic dysfunction of β-pancreatic cells and may favor the onset of DM [56].

MMPs and tissue inhibitors of metalloproteinases (TIMPs) are endopeptidases involved in morphogenesis, physiological tissue turnover, and pathological tissue destruction of the extracellular matrix (ECM). Currently, MMP-8 in oral fluids is a highly utilized marker in diagnosing periodontitis, while salivary MMP-9 serves as a marker of inflammatory status [57]. MMP-13, a collagenase found in fibroblasts, macrophages, osteoblasts, plasma cells, and gingival epithelial cells, is implicated in the destruction of periodontal soft tissue and, together with MMP-9, in bone resorption in periodontitis [58]. Bacterial load leads to the degradation of the extracellular matrix, enamel, and dentin through the activation of MMPs, especially MMP-2, MMP-9, and MMP-20.

The bidirectional relationship between DM and periodontitis is based on chronic inflammation, with each of them exacerbating the other [59]. Studies show that hyperglycemia leads to the increased activity of MMPs and oxidative stress. The determination of salivary MMP-9 levels in patients with periodontitis, with or without DM, shows values twice as high in the DM–periodontitis association compared to the group of patients with periodontitis alone [60]. A study conducted on diabetic patients with diabetic foot ulcers shows that elevated serum levels of MMP-9 and the MMP-9/TIMP-1 ratio are associated with poor wound healing [61].

The importance of epigenetics here is that the key proinflammatory molecules produced in periodontal disease can inflict epigenetic modifications, such as DNA or histone methylation and miRNAs, thereby deregulating the gene expression of both tissue and blood-based cells in favor of a greater systemic inflammatory milieu, with periodontal inflammation hypersensitizing the immune system towards additional insults.

Li et al. (2023) showed that a reduction in the expression of the histone deacetylase SIRT6 in macrophages, from both a murine model and human diabetic periodontitis, blocked the resolution of inflammation and increased the generation of apoptotic neutrophils, implicating epigenetic modulation in immune cell dysregulation [27].

Seutter et al. (2020) provided a detailed analysis of inflammation’s impact on periodontal gingival cells. Their study exposed human gingival fibroblasts to the key proinflammatory cytokine IL-1β in vitro, revealing a significant increase in DNMT-1 expression, alongside a decrease in DNMT3a and the demethylating enzyme TET1. Similarly, prostaglandin E2 (PGE2) induced comparable changes, effectively altering the cellular methylation pattern and upregulating gene transcription. This process contributed to the persistence of both local and systemic inflammation [62].

MMPs play a crucial role in inflammation by degrading the extracellular matrix (ECM) and promoting immune cell hyperplasia, thereby intensifying inflammatory processes. The hypermethylation of MMPs, such as MMP-9, has been shown to enhance this activity, making it a key factor in chronic and severe periodontitis [63,64].

More recently, Favale et al. (2024) used shotgun metagenomic analysis to confirm the bidirectional relationship between periodontitis and diabetes mellitus (DM) in promoting chronic endotoxemia and inflammation. Their findings highlight the role of epigenetic reprogramming and immune cell phenotype shifts originating in dysbiotic regions, leading to a self-sustaining disease microenvironment in both conditions [65].

## 3. Could Epigenetic Modulators Protect Against Cyclical Pathological Amplification Between PD and DM?

Recent evidence has shown that pathogenic systemic inflammatory and immunomodulatory effects seen in periodontitis correlate with premature onset and/or the worsening of chronic, serious vascular diseases such as CVD, ischemic stroke, and Alzheimer’s disease [66,67].

The two-way cause–effect relationship between periodontitis and DM has been previously discussed, but new evidence also indicates the significant role of epigenetic changes caused by periodontitis. For example, if these changes occur through miR-linked epigenetic regulation, they could lead to vascular endothelial cell dysfunction associated with comorbidities like hypertension and inflammation. Such mechanisms are major contributors to immunological changes predicting pathological progression in DM. Therefore, hypothetically, these same epigenetic pattern modifications could form the basis of future pharmacological protection against the PD-DM milieu [68].

Bascones-Martinez and González-Febles first described epigenetic changes in periodontitis as an independent risk factor for DM [69]. The genetic link between the specific gene polymorphism seen in periodontitis and the increased risk of DM was presented by Cao et al. [70], whilst Lee et al. identified the specific transcriptional changes in the monocytes of patients with periodontal disease activating the RESISTIN pathway, which strongly associate with increased insulin resistance and susceptibility to the development of DM [71]. A very recent study by Kang et al. carried out the whole-genome methylation profile in periodontitis patients using the single-cell RNA sequencing of peripheral blood monocytes. They showed a specific increase in hypomethylated genes associated with Fc-gamma receptor-mediated phagocytosis, correlating the same epigenetic changes in the pancreatic tissue with the same patients who also presented with DM [72].

In other conditions, such as in cancer therapy, epigenetic mediators, modifiers, or modulators are successfully used for their proapoptotic effect, and, in addition, the anti-inflammatory/immune-suppressive nature of inhibitors of DNMTs, HDACs, and bromodomain-containing proteins (BETs) indicates the potential use in the treatment of many other chronic diseases [73,74]. However, their use in the treatment of periodontitis, even if in much lower doses, is hypothetical, and limited by toxicity and side effects, such as thrombocytopenia, neutropenia, fatigue, and diarrhea [11].

### 3.1. Natural Potential Epigenetic Modulators in Chronic Disease

In this context, plant-based epigenetic modulators offer several advantages. Besides their lower toxicity, they possess multipotent properties, including antioxidant effects from compounds such as vitamins and polyphenols. Certain active principles also inhibit DNA methyltransferases (DNMTs), such as (–)-epigallocatechin-3-gallate (from *Camellia sinensis*), curcumin (from *Curcuma longa*), and quercetin, a dietary flavonoid found in many fruits and vegetables. Additionally, resveratrol—present in grapes, berries, soybeans, pomegranates, and peanuts—is produced by spermatophytes in response to injury and has notable epigenetic effects. Other compounds, such as genistein (from *Genista tinctoria* and *Glycine max*) and boswellic acids (from *Boswellia serrata*), promote the generation of S-adenosyl methionine (SAM) due to their folic acid content, which enhances Nrf2 expression by demethylating its promoter. Furthermore, several plant-derived histone deacetylase (HDAC) inhibitors contribute to epigenetic regulation. These include trichostatin A (from *Streptomyces hygroscopicus*), depudecin (from *Alternaria brassicicola*), and diallyl disulfide (from the *Allium genus*) [75,76].

These natural compounds not only modulate epigenetic mechanisms but also exhibit anti-inflammatory, antioxidant, and anticancer properties, making them promising candidates for therapeutic applications. For instance, curcumin has been identified as a highly potent HDAC inhibitor which is more efficacious than well-characterized HDAC inhibitors like valproic acid and sodium butyrate. Similarly, resveratrol and genistein have been shown to influence DNA methylation and histone modifications, thereby regulating the gene expression involved in various pathological conditions. The multifaceted actions of these phytochemicals underscore their potential as safer alternatives to synthetic epigenetic drugs, warranting further research into their mechanisms and therapeutic efficacy [77,78,79].

### 3.2. CBD as a Novel Epigenetic Modulator of the Periodontitis–DM Axis

Evidence has shown a strong protective effect of CBD within the periodontal tissue exposed to pathogenic bacteria. Specifically, CBD improves normal hemostatic activity, is osteoprotective (reducing bone resorption), and is strongly anti-inflammatory and bacteriostatic (e.g., antibiotic-resistant *Staphylococcus*) [80]. CBD is a natural ligand of the CB1 (antagonist) receptors, which are found in the brain, and CB2 (partial agonist) receptors, which are found at the cellular level, thus being responsible for anti-inflammatory and immunomodulatory effects. For example, in vivo and in vitro studies in human periodontal ligament fibroblasts show that CBD blocking CB2 activation inhibited inflammatory cytokine production (IL-1β and TNF-α) through TLR4-NF-κB signaling pathways and oxidative stress via the activation of PPAR-γ and Nrf2 [17,81].

**Figure 1:**
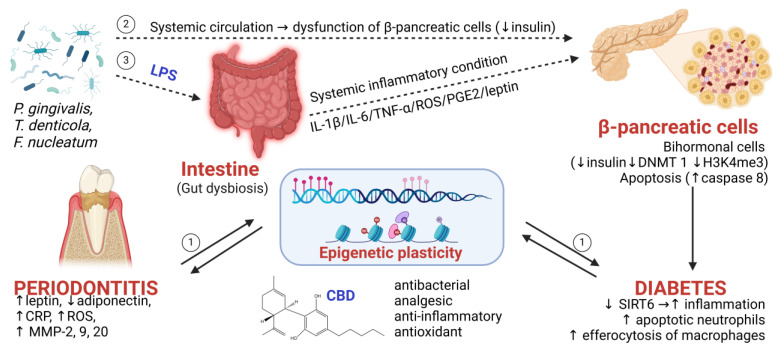
CBD as epigenetic modulator of chronic inflammatory conditions. ① Periodontitis, through the local inflammatory markers produced, generates epigenetic plasticity that is responsible for the appearance of systemic inflammatory diseases. Diabetes is a systemic inflammatory disease characterized by the occurrence of numerous complications, such as periodontitis by epigenetic modulation. The continuous lines suggest a well-known and accepted process based on numerous studies. ②, ③ The dotted lines suggest the proposed mechanisms by which Gram-negative bacteria in the oral cavity would be responsible for amplifying a local inflammatory process (periodontitis) to a systemic inflammatory process (diabetes) (C-reactive protein (CRP), reactive oxygen species (ROS) matrix metalloproteinases (MMP-2, 9, and 20), histone deacetylase sirtuin-6 (SIRT6), cannabidiol (CBD), *Porphyromonas gingivalis* (*P. gingivalis*), *Treponema denticola* (*T. denticola*), *Fusobacterium nucleatum* (*F. nucleatum*), interleukins (IL-1β, IL-6), tumor alpha (TNF), and prostaglandin E2 (PGE2)). This image was created using Biorender (https://www.biorender.com, accessed on 30 September 2024).

As a key modulator of inflammation, a first study performed on human keratinocytes in 2013 by Pucci et al. showed that CBD produced an increase in the global level of methylated DNA by increasing the activity of maintenance DNMT-1 [82]. Cannabinoids also demonstrated increased DNA methylation, the expression of nuclear transcription factor STAT6, and the production of a more anti-inflammatory phenotype in an in vivo model of atopic dermatitis in dogs. The authors further showed a mechanism involving the downregulation of the gene expression of ccl2, ccl17, and tslp in keratinocytes and of ccl2, ccl17, and il31ra in monocytes [83]. These observations indicated that proinflammatory gene expression was controlled primarily by the modulation of the epigenetic cell profile; hence, since the pathways are essentially overlapping, CBD should be epigenetically protective in periodontal disease (Figure 1) [84].

DNA hypomethylation in the prefrontal cortex has also been incriminated in anxiety-like behaviors, and recent studies have confirmed that the basis of the anxiolytic effects of CBD was an increase in the de novo DNMT3 activity, associated with reduced inflammation and a critical mediator associated with neuropsychological disturbance [14,85]. CBD reduced the DNMT activity in male Swiss mice, and exerted a serotonin-5-HT1A-dependent antidepressive effect when examined during the forced swimming test [86]. CBD epigenetically controlled the immunosuppressive effects on T-cells and macrophages, which occurred at the same time as the decreased production of TNF-α and interferon gamma (IFN-γ), in addition to the well-described antiapoptotic mechanisms in cancer cells [87].

CBD was also shown to modify cellular epigenetic profiles through the inhibition of the ten-eleven translocation methyl cytosine dioxygenase 1 (TET1) protein, an Fe (II) dioxygenase that transforms 5-methylcytosine to 5-hydroxymethylcytosine, and which regulates immune cell response and associated inflammation [88]. This inhibition is due both to the chelating effect of CBD against the Fe (II) ion and also to other types of interactions (e.g., hydrophobic interactions and hydrogen bonding) [89].

Despite its potential therapeutic applications, no major adverse effects of cannabidiol (CBD) have been reported so far. However, studies on cannabis use and sperm quality in humans and rats have revealed DNA hypermethylation, which may contribute to the transgenerational transmission of epigenomic instability [90]. Additionally, prenatal exposure to CBD has been linked to sex-specific changes in working spatial memory and anxiolytic behavior in rat offspring [91]. To minimize unwanted side effects, modifying CBD synthetically or combining it with other active compounds may be beneficial. For instance, a combination of moringin and CBD demonstrated an antiapoptotic effect by reducing Bax, Bcl-2, and caspase-3 levels in vitro. This combination also activated the PI3K/Akt/mTOR pathway, prolonging the survival of periodontal ligament mesenchymal stem cells—an essential factor in regenerative medicine [92,93]. Moreover, metformin has recently been recognized for its strong anti-inflammatory properties and epigenetic regulatory effects. It supports cellular protection through mechanisms such as autophagy and senescence control, including the modulation of SIRT1 and AMPK gene expression [94]. Given its role as a gold-standard diabetes treatment, combining metformin with epigenetic protectors like CBD could lead to more effective therapeutics, a concept explored in the following section.

### 3.3. Metformin Plus CBD as a Novel Synergistic Therapeutic

Metformin-associated epigenetic modulation involves the inhibition of histone deacetylases (HDACs) and the regulation of DNA methylation, which downregulates proinflammatory cytokines such as TNF-α, IL-6, and NF-κB. Additionally, metformin enhances autophagy via mTOR inhibition, reducing oxidative stress and preventing further periodontal damage, and, in addition, protecting the junctional endothelium against senescence, which was associated with hyperglycemia in a diabetic mouse model [95].

Through separate mechanisms, CBD contributes to epigenetic modulation by influencing microRNA (miRNA) expression and histone modifications. Through its interactions with CB1 and CB2 receptors, CBD was shown to suppress NF-κB and Toll-like receptor 4 activity, leading to decreased inflammatory signaling and reduced bone resorption in a rat model of LPS-induced periodontitis [81]. The combination of metformin and CBD may therefore provide a stronger anti-inflammatory and tissue-protective effect in periodontitis by simultaneously targeting different but interconnected epigenetic pathways.

In addition, insulin resistance, a key driver of type 2 diabetes (T2D), is closely linked to chronic low-grade inflammation and epigenetic alterations affecting glucose metabolism and insulin signaling pathways. Metformin acts primarily through AMPK activation, leading to improved glucose uptake, fatty acid oxidation, and reduced hepatic glucose production, in addition to downregulating receptors used for advanced glycation end-product activity; for example, in murine bone marrow-derived macrophages, where it promoted M2 polarization concomitant with IL-10 transcription [96]. Additionally, metformin influences DNA methylation patterns in gluconeogenic genes, contributing to its insulin-sensitizing effects, as reviewed by Agius et al. (2020) [97]. Meanwhile, CBD’s ability to modulate PPAR-γ activity enhances the glucose metabolism and insulin sensitivity, demonstrated both in vitro and in vivo in a case-controlled study following the treatment of neuropsychosis and the examination by contrast-enhanced positron emission tomography [98], while also protecting pancreatic β-cells from oxidative damage and inflammation-associated apoptosis in a type 1 murine streptozotocin-induced diabetic model [99]. Furthermore, treatment with CBD oil has been shown to suppress cytokine-induced insulin resistance and reduce plasma triglyceride levels by reducing IL-1β, IL-6, and TNF-α levels in a rat obesity model [100]. Therefore, when combined, metformin and CBD should exert a considerable beneficial synergistic metabolic effect, improving insulin signaling, reducing oxidative stress, and enhancing mitochondrial function, thereby offering a novel therapeutic avenue for insulin resistance.

Recent evidence suggests that the gut microbiome plays a crucial role in both periodontal health and metabolic disorders. Dysbiosis in the gut can lead to increased gut permeability, promoting systemic inflammation that exacerbates both insulin resistance and periodontal disease. Metformin has been shown to alter the gut microbiota composition by increasing beneficial bacteria, such as *Akkermansia muciniphila*, which enhances the gut barrier integrity, reduces endotoxemia, and inhibits the production of inflammatory cytokines such as IL-6 [101]. A study by He KY et al. (2023) identified a significant reduction in *Akkermansia muciniphila* in both irradiated mice and human patients undergoing radiotherapy, negatively correlating with diarrhea duration. Supplementation with these bacteria mitigated radiation-induced damage by producing propionic acid, which activated G-protein-coupled receptor 43 (GPR43) on intestinal epithelial cells. This increased histone acetylation, enhancing the expression of tight-junction proteins (occludin and ZO-1) and mucins, thereby strengthening the intestinal barrier and reducing damage. When metformin was added, a further increase in the bacterial concentration was achieved, thereby protecting against radiation-induced intestinal injury and systemic inflammation [102].

CBD, on the other hand, has anti-inflammatory effects in the gut, potentially lowering systemic inflammatory markers that contribute to both periodontitis and insulin resistance. For example, in a double-blind placebo-controlled clinical trial, Couch DG et al. (2023) showed that the application of CBD reduced inflammation in colonic mucosa by protecting claudin-5 expression, blocking PPAR-α, and controlling lactulose and mannitol absorption. Hence, by modulating gut microbiota diversity and integrity, the combination of metformin and CBD may provide additional benefits in reducing inflammation and metabolic dysfunction [103].

Hence, the combination of metformin and CBD offers a promising multi-targeted approach for managing periodontitis and insulin resistance by regulating epigenetics, metabolism, and immune balance. Metformin enhances autophagy, insulin sensitivity, and mitochondrial function, while CBD reduces inflammation, oxidative stress, and supports gut health. Together, they may lower chronic inflammation, improve metabolic homeostasis, and aid periodontal tissue regeneration. However, further clinical studies are needed to determine the optimal dosing, long-term efficacy, and safety. This combination holds potential for the integrated management of metabolic and inflammatory diseases.

Combining cannabidiol (CBD) with metformin requires careful consideration due to potential interactions that may affect blood sugar control and liver function. Both CBD and metformin can lower blood glucose levels through different mechanisms. When taken together, there is a possibility of an additive effect, leading to hypoglycemia (low blood sugar). Symptoms of hypoglycemia include dizziness, fainting, and confusion. To mitigate this risk, it is advisable to monitor blood sugar levels closely and consult a healthcare provider before combining these substances. CBD is metabolized by liver enzymes, particularly the cytochrome P450 [104]. Concurrent use of CBD with other medications processed by these enzymes, such as metformin, may alter their effectiveness or increase the risk of side effects. Additionally, combining CBD with medications that can affect the liver, like pioglitazone (often combined with metformin), which may heighten the risk of liver problems. Signs of liver issues include fatigue, nausea, abdominal pain, dark urine, and jaundice (yellowing of the skin or eyes). If any of these symptoms occur, seek medical attention promptly.

CBD inhibits cytochrome P450 enzymes, which can affect the drugs that rely on these enzymes for metabolism. However, metformin is not metabolized by CYP enzymes, so direct competition is unlikely. Studies, including that of Zhu HJ et al. (2006), showed that CBD can alter gut absorption by affecting transport proteins like P-glycoprotein (P-gp), which could theoretically impact metformin absorption, but the evidence is limited to a few sporadic publications. There is no strong evidence showing that CBD directly reduces metformin’s bioavailability, but individual variations exist. CBD may influence metformin absorption or clearance due to its effects on transport proteins. Close monitoring and medical supervision are advised when using both together [105].

Although there is currently no official regulatory approval for using CBD and metformin together, they can be taken concurrently under medical supervision. While metformin is a prescription drug, CBD is widely available over the counter, though its effects on drug metabolism warrant caution. Doctors can prescribe both off-label, but, if a pharmaceutical company wanted to develop a combined therapy, it would require clinical trials and approval from regulatory agencies like the FDA or EMA. Patients should consult healthcare providers to monitor potential interactions, especially regarding blood sugar control and liver function.

General limitations: Despite its promising therapeutic potential, CBD faces significant limitations in clinical application due to its poor bioavailability, variable pharmacokinetics, and lack of standardized formulations. CBD is highly lipophilic and undergoes extensive first-pass metabolism in the liver, leading to low and inconsistent plasma concentrations when administered orally. This makes it difficult to establish precise dosing guidelines, which are essential for achieving consistent therapeutic effects across different patient populations. Additionally, the variability in CBD’s pharmacokinetics—depending on the formulation (oil, capsules, or inhaled)—further complicates its clinical use, making it challenging to develop reliable treatment protocols comparable to conventional therapies.

Another critical limitation is the lack of comprehensive safety data and regulatory oversight. While preclinical and early clinical studies suggest a favorable safety profile, concerns remain regarding potential drug interactions, particularly with medications metabolized via the cytochrome P450 enzyme system. This raises concerns about hepatic toxicity and altered drug metabolism, which can affect the efficacy and safety of concurrent treatments. Additionally, the long-term effects of chronic CBD use remain poorly understood, particularly in populations with underlying metabolic disorders, such as diabetes. The absence of large-scale clinical trials evaluating its effectiveness compared to conventional anti-inflammatory and immunomodulatory agents further limits its integration into mainstream medicine. Without standardized dosing regimens, validated clinical protocols, and long-term safety assessments, CBD’s full potential as a therapeutic agent remains uncertain

## 4. Materials and Methods

**Figure 2:**
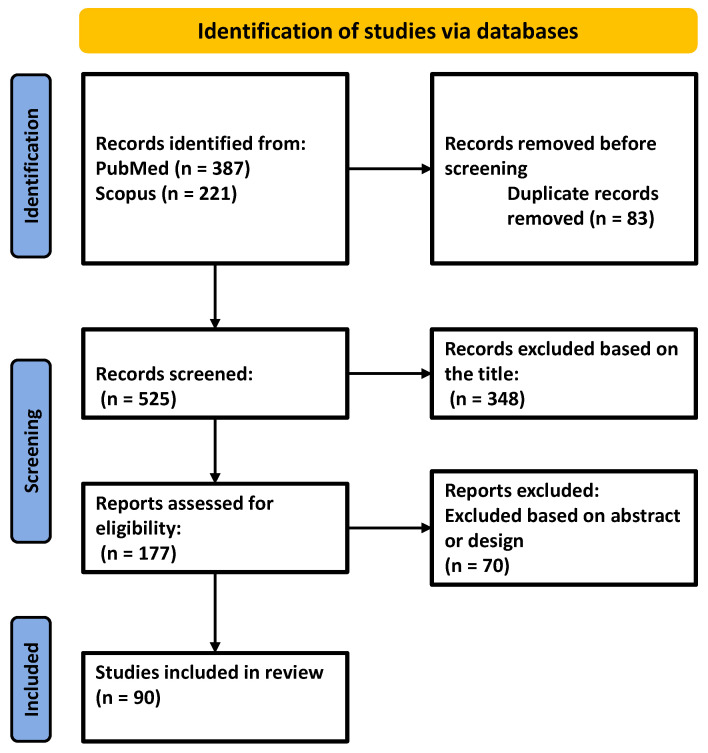
Flow diagram of the literature review process; n = number of articles.

For this narrative review, a systematic search on Scopus and PubMed was performed using combinations of the following keywords: “periodontitis”, “diabetes”, “inflammation”, “cannabidiol”, and “epigenetics”. EndNote 9 software was used to collect the articles published in the last 10 years (2014–2024), and, after removing duplicates, screening the title, abstract, and study design, the articles were retained for the narrative review (Figure 2). Non-English articles as well as gray literature were excluded. Additionally, articles unrelated to the periodontitis or changes associated with acute or traumatic conditions were excluded based on their title, abstract, and study design. Review articles as well as original articles of research, both preclinical and clinical studies, written in English, were included, with an emphasis on the latest published articles guided by the experience of the authors. Following this selection, 90 articles remained and were included in the present review.

## 5. Conclusions

Experimental molecules such as CBD have already shown promise as a potential therapeutic in the treatment of certain chronic diseases, for example, the effective control of anxiety and depression through the regulation of cortical histones by CBD in rats via the trimethylation of H3K4me3, which was associated with transcriptional repression. In animal models of diabetes (types 1 and 2), CBD has demonstrated many protective effects, including a reversal of all major symptoms and insulin resistance, as well as a reduction in ensuing comorbidities, such as atherosclerosis and CVD. To date, the only effective treatment for periodontitis is the use of local and/or systemic antibiotics (non-surgical), and so protective and preventative therapeutics are sorely needed. Although there are zero studies, to our knowledge, reported in the literature regarding the therapeutic benefits of epigenetic modulation in periodontitis, the anti-inflammatory properties demonstrated within the oral cavity by numerous studies, in conjunction with the critical epigenetic modulatory effects shown in other chronic diseases, make CBD, together with other potentially re- or dually purposed drugs (e.g., metformin), important candidates in the fight against periodontitis and associated comorbidities such as dementia, as a predisposing factor in DM, and directly as a protective factor against the development of DM.

## Figures and Tables

**Table 1:** Epigenetic modulation linking inflammation through the periodontitis–DM axis.

General Mechanism	Study Type	Administration Pathway	Mechanistic Details	Reference
Gut dysbiosis induced by oral bacteria	In vivo, C57BL/6 mice	Ligature-induced periodontitis + the administration of *P. gingivalis* by gavage	High activity of DNMT3b in gut and maxilla tissue; Gut dysbiosis induced by the dissemination of periodontal pathogens.	[22]
	In vitro, human osteoblast hFOB1.19 cells	0, 2, 5, or 10 µg/mL LPSs	The miR-124-3p pathway, with the systemic overexpression of DNMT3b resulting in excessive DNA methylation of the TRAF6 promotor; Increased inflammation via TNF-α and IL-6; Reduced cellular viability and increased apoptosis.	[23]
β-cell apoptosis and β-pancreatic bi(poli)-hormonal cells	In vivo, C57BL/6 mice	22 weeks oral application of *P. gingivalis*	Complex alterations in pancreatic islet morphology; Hyperinsulinemia mediated by SerpinE1, leading to insulin resistance and prediabetes; Stressed β-cell apoptosis associated with *P. gingivalis* activation of caspase-3 and 8, and reduced protection via the inhibition of the Akt pathway.	[24]
	In vivo, NKX2.2^ΔBeta^ mice	-	Gut pathogens decreased DNMT-1 activity, responsible for the production of β-cell-specific transcription factors PDX1 and NKX2.1; NKX2.2 is responsible for the maintenance of a functional, monohormonal β-cells by directly activating β-cell genes and actively repressing genes that induce modifications; The appearance of β-pancreatic bi-hormonal cells that lack the ability to produce insulin and secrete other hormones.	[31,33]
Epigenetic plasticity responsible for the inducement and dissemination of inflammation	In vitro/in vivo (murine model)	Ligature ligation and oral infection with *P. gingivalis*	*P. gingivalis* initiates innate (macrophages/polarization, dendritic cells, natural killer cells, monocytes, and neutrophils) and adaptive (B- and T-lymphocytes) immune responses; Periodontal cells make temporary changes to their phenotype for the resolution of inflammation; The production and release of proinflammatory molecules (I-IFN-γ, IL-17, TNF, IL-1, and IL-6) and associated enzymes (MMPs); Local proinflammatory mediators enter the systemic circulation, affecting other organs such as the pancreas.	[3,26,34]
	In vitro, CD105-enriched periodontal ligament-derived mesenchymal stem cells	*P. gingivalis* total protein extract (PgPE) (0 or 2 ug/mL) for 3 h	PgPE increases the secretion of the following inflammatory markers: RANTES, eotaxin, IFN-γ- inducible protein 10 (IP-10), monocyte chemoattractant protein-1 (MCP-1), IFN-γ, IL-6, IL-8, and IL-1ra.	[35,36]
	In vitro, Mouse periodontal ligament fibroblasts (mPDLFs)	LPSs (0.5 mM, 1 mM, and 2 mM)	LPS induces the NLRP3 inflammasome, caspase-1 activation, and the production of IL-1β.	[37]

## Abbreviations

The following abbreviations are used in this manuscript:

BETs	bromodomain-containing proteins
CAT	catalase
CBD	cannabidiol
CRP	C-reactive protein
CVD	cardiovascular disease
DM	diabetes mellitus
DNMT-1	DNA methyltransferase-1
DNMT3b	DNA methyltransferase-3 beta
DNMTs	DNA methyltransferases
ECM	extracellular matrix
HATs	histone acetyltransferases
HDACs	histone deacetylases
HFD	high-fat diet
hPDLSCs	human periodontal ligament stem cells
HSC	hematopoietic stem cell
IHC	immunohistochemistry
ILs	interleukins
IRS-1	insulin receptor substrate-1
LPSs	lipopolysaccharides
MDA	malondialdehyde
MMPs	matrix metalloproteinases
NAD+	nicotinamide adenine dinucleotide
NLRP3	NOD-like receptor family pyrin domain-containing 3
Nox2	NADPH oxidase 2
OPG	osteoprotegerin
PDLFs	periodontal ligament fibroblasts
PGs	prostaglandins
PPAR-α	peroxisome proliferator-activated receptors alpha
RANKL	receptor activator of NF-κB ligand
SIRT6	sirtuin-6
SOD	superoxide dismutase
TAOC	total antioxidant capacity
TET1	ten-eleven translocation methyl cytosine dioxygenase 1
THC	Δ9-tetrahydrocannabinol
TIMPs	tissue inhibitors of metalloproteinases
TNF-α	tumor necrosis factor alpha
TRAF6	tumor necrosis factor receptor-associated factor 6
TXNIP	thioredoxin
